# Localisation osseuse sternale et claviculaire d'une amylose

**DOI:** 10.11604/pamj.2014.19.94.5008

**Published:** 2014-09-26

**Authors:** Neirouz Ghannouchi Jaafoura, Amira Atig

**Affiliations:** 1 Service de Médecine Interne, CHU Farhat Hached, Sousse, Tunisie

**Keywords:** Déminéralisation osseuse, lésion ostéolytique, amylose, bone demineralization, osteolytic lesion, amylosis

## Image en medicine

Un homme de 46 ans, sans antécédents pathologiques notables, est hospitalisé pour une altération de l’état général avec troubles de la marche évoluant depuis 6 mois. On constate à l'examen physique des ecchymoses périorbitaires droites et une tuméfaction de l'extrémité interne de la clavicule droite et du sternum. L'imagerie standard ainsi que la tomodensitométrie thoraco-abdominale révèle un aspect hétérogène diffus à toute la trame osseuse avec une fracture pathologique de l'extrémité externe de la clavicule (A) et une masse lytique sternale (B) dont la biopsie conclu à la présence de dépôts amyloïdes. On notait par ailleurs à la biologie une hypercalcémie à 2,8 mmol/l et une anémie normochrome normocytaire à 10 g/l. L’électrophorèse des protides était normale et le myélogramme ne montre pas d'infiltration par des plasmocytes dystrophiques. A La biopsie ostéomédullaire on note la présence d'une amylose médullaire et à l’échographie cardiaque, un aspect infiltré tigré du myocarde, un trouble restrictif et une lame d’épanchement péricardique. L’évolution était rapidement fatale avant le typage de l'amylose. La localisation osseuse de l'amylose, révélatrice chez ce patient, est rare. Elle réalise un tableau clinique de tuméfaction osseuse ou de fracture pathologique avec atteinte essentiellement vertébrale. Le principal diagnostic différentiel est celui de lésion tumorale maligne primitive ou secondaire.

**Figure 1:**
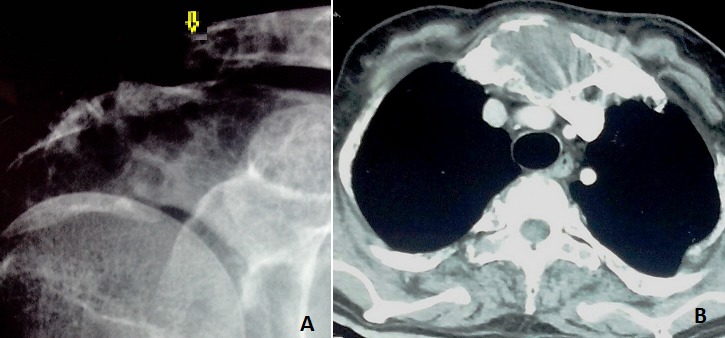
A) radio de l’épaule vue de face: déminéralisation osseuse avec fracture de l'extrémité externe de la clavicule; B) Scanner thoracique: lésions ostéolytiques du sternum et du corps vertébral

